# Multifocal Post-Traumatic Myositis Ossificans Circumscripta in a Young Male Following a Motor Vehicle Accident: A Review of Imaging and Clinical Presentation

**DOI:** 10.7759/cureus.14328

**Published:** 2021-04-06

**Authors:** Ahjay Bhatia, Ashley Ryan Vidad, Divy Mehra, Himadri Shah, Oluwaseun Ogunjemilusi

**Affiliations:** 1 Osteopathic Medicine, Nova Southeastern University Dr. Kiran C. Patel College of Osteopathic Medicine, Plantation, USA; 2 Family Medicine, Internal Medicine, Surgery, Pathology, Gynecology, Infectious Disease, Neurology, Orthopedics, Gastroenterology, Nova Southeastern University Dr. Kiran C. Patel College of Osteopathic Medicine, Fort Lauderdale, USA; 3 Ophthalmology, Nova Southeastern University Dr. Kiran C. Patel College of Osteopathic Medicine, Fort Lauderdale, USA; 4 College of Osteopathic Medicine, Nova Southeastern University Dr. Kiran C. Patel College of Osteopathic Medicine, Davie, USA; 5 Internal Medicine, Westside Regional Medical Center, Plantation, USA

**Keywords:** radiology, myositis ossification, post trauma, radiological findings, radiological diagnosis, bone pathology, non-surgical orthopedics, trauma imaging, anatomic imaging, myositis ossificans

## Abstract

Traumatic myositis ossificans (MO) circumscripta is an uncommon nonhereditary pathophysiological result of muscular trauma that is detected by radiographic imaging three to four weeks following initial trauma. It is responsible for great global morbidity, with symptoms of prolonged pain, diminished flexibility, and stiffness. There is frequently a delay in diagnosis due to the generalized symptoms and varying radiographic presentation. The goal of therapy is to rule out serious complications (such as soft tissue sarcoma) and to restore strength and range of motion (ROM) as soon as possible. Here we detail the case of a 32-year-old male with a delayed diagnosis of MO who presented to the hospital with left lower extremity pain and swelling following a motor vehicle accident (MVA) that occurred one month prior.

## Introduction

Traumatic myositis ossificans (MO) circumscripta is an inflammatory disorder that arises in skeletal muscle [1]. It is a benign, solitary, self-limiting disorder representing metaplasia of bone. Traumatic MO circumscripta represents one of three distinct disorders that are all characterized by the development of ectopic bony tumors in different skeletal muscle locations: MO progressive, traumatic MO circumscripta, and MO circumscripta without history of trauma [2]. The pathophysiology of MO circumscripta formation is not completely understood but is thought to occur through differentiation of fibroblasts into osteogenic cells [2]. Kan et al. [3] demonstrated that the cellular mechanism of heterotopic bone formation is the result of the dysregulation of local stem cells in response to tissue injury and inflammation. Men and women are equally affected with no preferential anatomical site, though clinical cases in the girdles and limbs are frequently described [1].

The diagnosis of MO is often difficult to make and regularly overlooked. Early MO without a considerable amount of calcification and ossification can lead to either false negative radiological readings or misdiagnosis as other, more serious conditions (osteosarcoma) [4]. A high level of clinical suspicion is necessary as complaints are nonspecific (e.g. pain, swelling) and diagnosis is predominantly radiological. Treatment is conservative, as the process tends to be self-limiting and self-resolving, and generally consists of nonsteroidal anti-inflammatory drugs (NSAIDs) and physical therapy [5-6]. Here, we detail the delayed diagnosis of traumatic MO circumscripta in a young male following a motor vehicle accident (MVA) to further highlight and raise clinical suspicion for the disease in post-traumatic patients. 

## Case presentation

A 32-year-old male with no chronic medical conditions presented to the ED complaining of worsening left lower extremity swelling and pain. One month prior to presentation, this gentleman suffered an MVA, presented to the ED, and was discharged after exclusion of a deep vein thrombosis (DVT).

During the current visit, the patient was afebrile, and his vitals were stable. On physical exam, the left lower extremity was edematous and warm to touch with normal pulses, no limitations in range of motion (ROM), no calf tenderness, no motor or sensory deficits, and no other neurologic abnormalities. A venous duplex ultrasound of both lower extremities was ordered and showed no sonographic evidence of DVT. Laboratory workup revealed abnormal renal function (estimated glomerular filtration rate, GFR of 39, blood urea nitrogen, BUN of 37 mg/dL, creatinine of 2.00 mg/dL), transaminitis [aspartate aminotransferase (AST) of 107 U/L], an elevated D dimer, mildly elevated parathyroid hormone (PTH), and mild decreased values of red blood cell (RBC) count (3.36*106/uL), hemoglobin (10.1 g/dL), and hematocrit (30.4%). A bilateral renal artery ultrasound was performed to check for signs of hydronephrosis but it was negative and showed increased echogenicity of the renal cortices, correlating clinically for chronic medical renal disease. Based on the clinical picture and laboratory findings, we made a diagnosis of mild acute kidney injury and rhabdomyolysis with secondary hyperparathyroidism and transaminitis. The subsequent plan was to administer pain control with acetaminophen 500 mg as needed, cholecalciferol (vitamin D3) 25 mcg daily, IV normal saline at 100 cc/h, and prophylactic antibiotics (azithromycin and ceftriaxone), as well as to consult nephrology.

**Figure 1:**
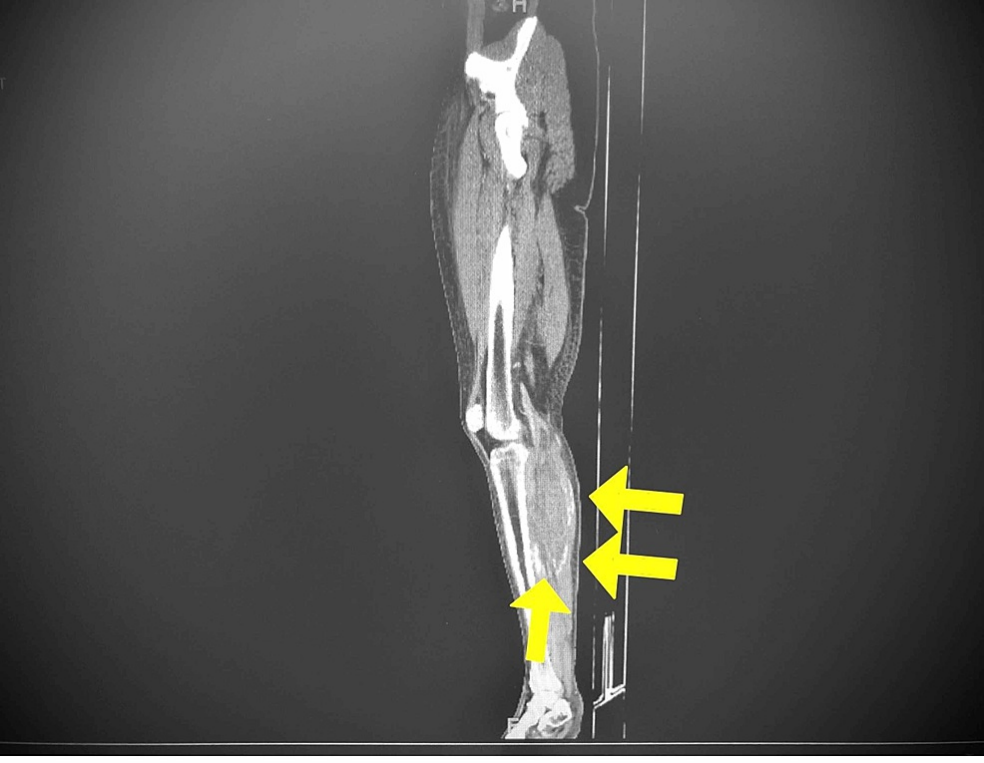
CT (sagittal view) of the left lower extremity. Curvilinear calcifications within the proximal lateral gastrocnemius and soleus muscles.

**Figure 2:**
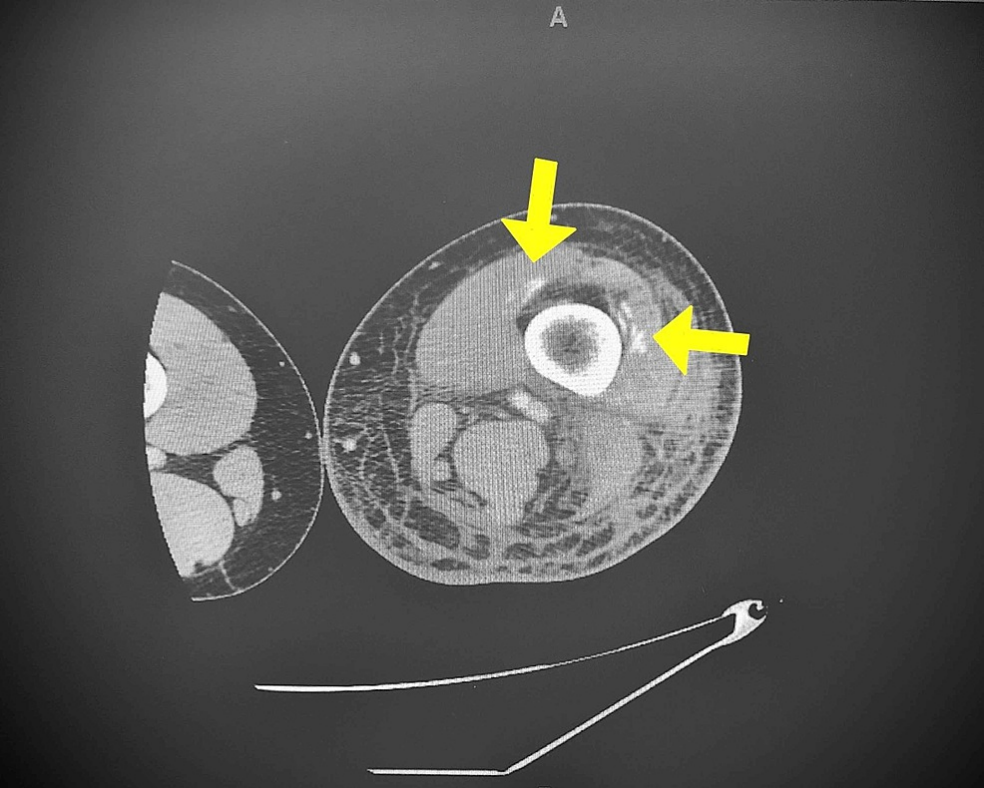
CT (axial view) of the left lower extremity. Curvilinear calcifications within the distal vastus intermedius, vastus lateralis, and vastus medialis.

Despite this hospital management, the patient’s leg pain and swelling progressively worsened and after three days a CT scan with contrast of the left lower extremity was ordered. The CT revealed subcutaneous and intrafascial edema (particularly in the anterior compartment of the proximal thigh); further, curvilinear calcifications within the distal vastus intermedius, vastus lateralis, and vastus medialis (Figure 1), proximal lateral gastrocnemius, soleus, fibularis longus (Figure 2), flexor hallucis longus, and tibialis posterior muscles. A cleft was noted between these calcifications and underlying bone. The overall impression was of MO involving the distal anterior compartment of the thigh and posterior anterior compartment of the lower leg, suspected as a result of prior trauma (MVA). An orthopedic consultation was organized for follow up in the outpatient setting and the patient was subsequently discharged after being administered lidocaine patches and discussing the avoidance of NSAIDs and aspirin until the resolution of renal issues.

## Discussion

Traumatic MO circumscripta is a benign, self-limiting condition characterized by heterotopic bone formation, usually involving striated muscle [5]. There is equal occurrence in both men and women with adolescents and young adults under 30 being the most commonly affected [7]. The diagnosis of MO is made clinically and radiologically.

Both the clinical presentation and radiological findings of MO vary and are correlated with the stage of disease progression of the patient [4]. The early stage (< 4 weeks) is characterized by diffuse pain and swelling and there is little to no osteoid formation [2]. These generalized symptoms make it difficult for clinicians to pick up MO early in the disease course, as well as for radiologists to identify the disease on imaging. For these reasons, most cases of MO are typically diagnosed three to four weeks after the initial trauma with MRI being the preferred modality [5]. The intermediate stage of MO (four to eight weeks) is characterized by immature osteoid formation, which gradually changes into mature bone on the periphery of the mass, and the mature stage (> 8 weeks) includes mature lamellar bone [8].

Radiographic findings of MO are important to recognize as they can often be confused with or misdiagnosed as more sinister conditions like osteosarcoma [9]. Peripheral, curvilinear calcifications (Figure 1) allow for distinction between the two pathologies, as central calcifications are more characteristic for bone forming sarcomas [9]. Additionally, the extensive muscle edema characteristic of the early stage of MO is unusual in most primary neoplasms [9]. Intralesional post-contrast enhancement, however, may lead the clinician to favor osteosarcoma, although such descriptions have been noted in past MO cases [10]. A misdiagnosis as a neoplasm can lead to unnecessary biopsy of the ossification and can worsen prognosis of MO [10-11].

Treatment of MO is supportive as the disease is self-limiting. NSAIDs and physical therapy are the mainstays of treatment to reduce swelling and increase ROM of the affected area, especially in cases involving the extremities [12]. Surgical intervention is only necessary when the patient has unbearable pain or when secondary complications (nerve impingement) arise [12]. In the current case, NSAIDs were avoided to a history of renal issues. Alternative analgesics like acetaminophen or opiates can be used, but NSAIDs are preferred when indicated due to their anti-inflammatory properties.

## Conclusions

Traumatic MO circumscripta can go undiagnosed or be misdiagnosed due to nonspecific symptoms on clinical presentation, as well as due to the inconsistent findings on imaging (a result of the progression of the disease). As such, it is important to have a high level of clinical suspicion in post-traumatic patients to catch the disease early in its disease course. 
